# Cancer Cluster Investigations: Review of the Past and Proposals for the Future

**DOI:** 10.3390/ijerph110201479

**Published:** 2014-01-28

**Authors:** Michael Goodman, Judy S. LaKind, Jerald A. Fagliano, Timothy L. Lash, Joseph L. Wiemels, Deborah M. Winn, Chirag Patel, Juliet Van Eenwyk, Betsy A. Kohler, Enrique F. Schisterman, Paul Albert, Donald R. Mattison

**Affiliations:** 1 Department of Epidemiology, Rollins School of Public Health, Emory University, 1518 Clifton Road, Atlanta, GA 30322, USA; E-Mails: mgoodm2@emory.edu (M.G.); timothy.lee.lash@emory.edu (T.L.L.); 2 LaKind Associates, LLC, 106 Oakdale Avenue, Catonsville, MD 21228, USA; 3 Department of Epidemiology and Public Health, School of Medicine, University of Maryland, Howard Hall Suite 200, 660 W. Redwood Street, Baltimore, MD 21201, USA; 4 Department of Pediatrics, College of Medicine, Milton S. Hershey Medical Center, Pennsylvania State University, 500 University Drive, Hershey, PA 17033, USA; 5 Division of Epidemiology, Environmental and Occupational Health, New Jersey Department of Health, P.O. Box 369, Trenton, NJ 08625, USA; E-Mail: Jerald.Fagliano@doh.state.nj.us; 6 Division of Cancer Epidemiology, Department of Epidemiology & Biostatistics, School of Medicine, University of California, Helen Diller Family Cancer Research Building, HD 274 1450 3rd Street, San Francisco, MC 0520, San Francisco, CA 94158, USA; E-Mail: Joe.Wiemels@ucsf.edu; 7 Division of Cancer Control and Population Sciences, National Cancer Institute, National Institutes of Health, 9609 Medical Center Drive, Bethesda, MD 20892, USA; E-Mail: WINNDE@MAIL.NIH.GOV; 8 School of Medicine, Stanford University, 1265 Welch Road, Stanford, CA 94305, USA; E-Mail: cjp@stanford.edu; 9 Washington State Department of Health, P.O. Box 47812, Olympia, WA 98504, USA; E-Mail: juliet.vaneenwyk@doh.wa.gov; 10 North American Association of Central Cancer Registries, Inc., 2121 W. White Oaks Drive, Suite B, Springfield, IL 62704, USA; E-Mail: bkohler@naaccr.org; 11 Division of Epidemiology, Statistics and Prevention Research, Eunice Kennedy Shriver National Institute of Child Health and Human Development, National Institutes of Health, 6100 Executive Blvd., Bethesda, MD 20892, USA; E-Mails: schistee@mail.nih.gov (E.F.S.); albertp@mail.nih.gov (P.A.); 12 Risk Sciences International, 325 Dalhousie Street, Ottawa, ON K1N 7G2, Canada; 13 McLaughlin Centre for Population Health Risk Assessment, University of Ottawa, 325 Dalhousie Street, Ottawa, ON K1N 7G2, Canada; E-Mail: mattisond@risksciences.com

**Keywords:** cancer, cluster investigations, cancer biomarkers, case grouping, leukemia, exposome, infection

## Abstract

Residential clusters of non-communicable diseases are a source of enduring public concern, and at times, controversy. Many clusters reported to public health agencies by concerned citizens are accompanied by expectations that investigations will uncover a cause of disease. While goals, methods and conclusions of cluster studies are debated in the scientific literature and popular press, investigations of reported residential clusters rarely provide definitive answers about disease etiology. Further, it is inherently difficult to study a cluster for diseases with complex etiology and long latency (e.g., most cancers). Regardless, cluster investigations remain an important function of local, state and federal public health agencies. Challenges limiting the ability of cluster investigations to uncover causes for disease include the need to consider long latency, low statistical power of most analyses, uncertain definitions of cluster boundaries and population of interest, and in- and out-migration. A multi-disciplinary Workshop was held to discuss innovative and/or under-explored approaches to investigate cancer clusters. Several potentially fruitful paths forward are described, including modern methods of reconstructing residential history, improved approaches to analyzing spatial data, improved utilization of electronic data sources, advances using biomarkers of carcinogenesis, novel concepts for grouping cases, investigations of infectious etiology of cancer, and “omics” approaches.

## 1. Introduction

Residential clusters of non-communicable diseases are a source of enduring public concern, and at times, controversy [1,2,3]. Compared to clusters in which cases are linked by common occupation such as working with asbestos in a cluster of mesothelioma [4], or share an unusual risk factor such as prenatal exposure to diethylstilbestrol in a cluster of clear cell carcinoma of the vagina [5], clusters that appear to arise in a given geographic area or in a given community are particularly difficult to study. 

Descriptions of non-occupational geographic clusters of cancer (primarily leukemia) can be found in the literature as far back as the beginning of the 20th century [6] and published systematic reviews of this issue span nearly 40 years [7,8]. Other diseases that have been reported to cluster in space and time include birth defects [9,10], autism [11,12,13], multiple sclerosis [14,15], amyotrophic lateral sclerosis [16,17] and suicide [18,19]. While a wide array of health outcomes have been reported to cluster, what sets cancer clusters—and especially pediatric cancer clusters—apart are the frequency with which they are reported and the existence of population-based cancer registries to readily and accurately identify cases in a defined geographical area. We therefore focus on cancer clusters in this paper, although much of the content would apply in equal measure to clusters of other diseases.

The published recommendations on how to conduct cluster investigations have remained largely unchanged over the last three decades. In 1981, Aldrich [20] proposed starting with a definition of the potential cluster event, followed by the determination of the population at risk, and then an assessment of whether further study is warranted. Once a full study is deemed necessary, the investigators would consider developing a study questionnaire aimed at testing “the battery of reported theories related to the specific disease etiology” [20]. 

In 1989, a National Conference on Clustering of Health Events summarized the preceding twenty years of experience of cluster investigations and discussed specific methodological features of such investigations [21,22,23,24,25]. In 1990, the Centers for Disease Control and Prevention (CDC) issued their Guidelines for Investigating Clusters of Health Events [26]. The CDC guidelines outlined a four-stage approach, which was similar to that proposed by Aldrich [20], and included the following components: initial contact and response (Stage 1); an assessment to confirm existence of a cluster (Stage 2); an evaluation of feasibility of a full scale epidemiologic study (Stage 3); and, if warranted, a formal etiologic investigation (Stage 4). 

In 2007, CDC issued an addendum to the 1990 guidelines by specifically addressing investigations of cancer clusters [27]. Among the criteria used to justify the move from initial to more complex stages of investigation were: a statistical excess of a single type of cancer; a rare cancer type; a common cancer in an unusual age group; or suspected exposure to a known carcinogenic agent with sufficient elapsed time since exposure [27]. The most recent CDC guidelines for cancer cluster investigations were issued in late 2013, and continued to recommend the previously adopted four-stage approach. In addition, the 2013 guidelines highlighted data sources and statistical techniques that could be used in cancer cluster investigations, and described possible approaches for developing effective communication strategies. The stated goals of these updated guidelines were “to provide needed decision support to public health agencies in order to promote sound public health approaches, facilitate transparency and build community trust” [28]. 

While goals, methods and conclusions of cluster studies are debated in both the scientific literature [8,25,29,30,31] and the popular press [32,33,34,35], investigations of reported residential clusters rarely provide definitive answers about disease etiology [8,36,37,38,39,40]. Further, it is inherently difficult to study a cluster for a disease with complex etiology and long latency such as most cancers. Despite this difficulty, evaluation of clusters remains an important function of local, state and federal public health agencies. Early and timely involvement of public health agencies is critical because a poor initial response can result in missed opportunities for an investigation and education and may increase the level of uncertainty and concern in a community, potentially resulting in the need to later expend additional public health resources.

Several recent reviews argued that progress in cluster research may require fundamental, rather than incremental, changes in methodology, and have recommended the development and testing of novel or previously understudied hypotheses [8,41,42,43,44]. The current communication summarizes deliberations of the multi-disciplinary two-day workshop “*Advancing Cancer Cluster Assessments: Starting the Dialogue*” held in April 2013 with the goal of advancing the search for new approaches to studying this issue. The workshop included researchers with specific relevant expertise in epidemiology, biostatistics, informatics, exposure science, clinical medicine, disease surveillance, and risk communication. Workshop participants came from a variety of settings, including federal and state public health agencies, academic and government research organizations, and the private sector. Although several participants had first-hand involvement in cluster investigations, the workshop did not focus on findings from previous studies, but rather used past experience to identify key issues that need to be considered in future cluster investigations. The results of the workshop discussions are described here. We first review definitions and goals associated with cancer cluster investigations, then describe investigation-related challenges, and finally describe novel or under-explored approaches that could potentially be added to the arsenal of current approaches for investigating clusters. It is the hope of the workshop participants that this communication will prompt those involved in various aspects of cancer cluster investigation (representatives of the community, health agencies, and academic research institutions) to consider new ways of thinking about this long-standing problem. 

## 2. What is a Cancer Cluster and What are the Goals of Investigating Clusters?

In its 1990 guidelines, the CDC defined a cluster as “…an unusual aggregation, real or perceived, of health events that are grouped together in time and space and that are reported to a health agency” [26]. CDC later sharpened the definition, in the context of cancer investigations, as “…a greater-than-expected number of cancer cases that occurs within a group of people in a geographic area over a defined period of time” [27,45]. This re-definition focuses on the cluster as a statistical excess in a specified population, geographic area, and time period, and is not dependent on its perception, reporting, or existence of a common cause. 

Many clusters reported to the public health agencies by concerned citizens are accompanied by an expectation that an investigation will uncover a specific environmental cause of disease in the affected community [3,30,37,46]. By this measure, with few exceptions, cancer cluster investigations have not been successful [8]. Public health authorities and researchers acknowledge that cluster investigations rarely find statistical associations between local factors and disease incidence, and further that these investigations cannot demonstrate causality [8,27,31,37].

However, while understanding the role of known or perhaps novel risk factors is an objective of cluster investigations, it may not be the only objective. Even if following an investigation the etiology of disease remains unclear, the report of a cluster by the community and the proposed link to a possible cause can sometimes bring to light public health, environmental, social or other problems that should and could be mitigated even if not directly related to the community-reported concern. Neutra [31] emphasized that, as “part of good, empathetic public health practice”, health agencies need to have trained staff to promptly respond to concerns about potential clusters, including assessment of disease occurrence as well as environmental factors of concern to the community. The 1990 CDC Guidelines [26] noted that “reports of clusters cannot be ignored,” and public health agencies should adopt a leadership role in responding to concerns that “maintains community relations…without excessively depleting resources”. The intention of CDC’s guidelines and many states’ cluster response protocols is to screen and prioritize reports to limit investigations to those most likely to produce meaningful results [27,37]. Similarly, Condon *et al.* [29] noted that health agencies have a responsibility to the public to respond to community concerns, and that interactions in the course of an investigation provide opportunities to educate an engaged group of citizens on the frequency, etiology, and prevention of cancer, as well as on exposure issues of concern. Further, without this engagement, health agencies might miss the rare instances where cancer cluster investigations using current methodologies might be productive. This engagement allows health agencies to address environmental issues or other locally important cancer-related factors, such as screening [29]. 

Thus, cancer cluster investigations may best be seen as the fulfillment of a health agency’s general mission to protect and improve health, rather than as a basic research program in the environmental etiology of cancer. However, in terms of advancing the basic (as opposed to applied) science of cancer etiology and prevention, researchers will remain interested in exploring clusters in terms of causality and those types of basic science explorations will most often fall outside the scope of health agency activities. 

## 3. Cancer Cluster Investigation Challenges

While a wide array of health conditions aggregate in space and time, cancer clusters present several unique challenges for the affected community and for health agencies and researchers. These challenges, which drive the need for continued thought on novel approaches for investigating cancer clusters, are briefly described here:

*Timing of disease development*: Most malignancies have induction periods measured in decades. Exceptions to this are cancers in infants and children (where by definition the induction period cannot be longer than months or several years), leukemias arising from radiation and chemotherapy treatments for certain cancers [47], and cancers in immunosuppressed organ transplant recipients [48,49]. This long induction period presents a particular challenge in investigations of residential cancer clusters [50], because even though current address is routinely collected in cancer registries, the complete residential history is usually not available. True geographic clusters may need to be defined by the co-localization of individuals many years prior to the cancer diagnoses. 

*Defining a* “*case*” *for inclusion*: Case identification and classification present additional problems in cancer cluster investigations. A reported cluster may comprise individuals with a very rare and histologically distinctive cancer such as glioblastoma multiforme [51]. However, most reports of cancer clusters include cases presenting with cancers of different organs that are not known to have a common etiology or common genetic basis. Attempting to determine a common underlying cause in this type of situation will likely produce a misleading result or no conclusive result. Further, even cancers that arise from the same organ and have the same International Classification of Disease (ICD) code (e.g., acute lymphoblastic leukemias) may represent different molecular types and have different etiologic mechanisms and should therefore not be viewed as a single group of cases [52]. 

*Problem of small numbers*: Sparsely populated geographic regions often experience wide year to year fluctuations in the number of cancer cases, leading to unstable estimates of cancer incidence. This impedes researchers’ ability to establish presence or absence of a cluster [37]. Small numbers of cases complicate the implementation of case control studies aimed at testing causal hypotheses, because these studies tend to lack statistical power and often produce measures of associations that are too imprecise to allow meaningful conclusions.

*Defining boundaries and cluster area populations*: The boundaries of perceived clusters are often based on social or neighborhood networks involving known cases rather than on the more relevant boundaries dictated by exposures of interest [53]. This misspecification may limit our ability to identify a cluster and to understand its etiology [54]. The result can be either failure to detect a true cluster (due to exclusion of potentially relevant cases) or observing a cluster where none exists (by excluding exposed disease-free individuals). 

*Migration*: Due to the long induction period between exposure to a carcinogen and development of disease, some exposed members of a population may no longer be living in the community where a cluster develops, resulting in under-counting of cases. Conversely, a case contributing to the overall cancer cluster may have been exposed to a carcinogen from an earlier exposure in a different geographic region resulting in over-counting of cases. In either situation, population movement in or out of a community may result in misclassification of exposure [55]. The effect of migration on cluster investigations may be particularly pronounced and difficult to assess if migrants and those who do not change residence differ with respect to socioeconomic, exposure, demographic or health-related characteristics [56]. 

*Challenges related to cancer registries*: Population-based cancer registries are the best source of data for measuring cancer burden in a geographic area and over time [57]. Cancer registries in the USA are certified annually by the North American Association of Central Cancer Registries based on the completeness, timeliness, and accuracy of data, which has contributed to highly standardized and reliable data. While these registries are fundamental to understanding the distribution of cancer in time and space, they do not currently contain all of the information necessary for investigating cancer clusters (e.g., residential history). As information reported to the registries comes exclusively from medical records, most data on personal behavioral risk factors or environmental exposures are not captured. Complete ascertainment of cancer cases can take up to two years from the date of diagnosis, due to local reporting laws and the complexity of the data [58]. For this reason, using registry data to confirm a reported excess of cancer cases can delay confirmation for up to two years. 

## 4. Proposed Novel or Under-explored Approaches for Investigating Cancer Clusters

The previously noted lack of success in identifying environmental risk factors through investigations of residential clusters indicates a need for fundamentally novel—rather than incrementally improved—approaches. Several novel or under-explored potentially productive approaches are described, each in different stages of development by academic researchers and/or health agencies. Each approach has advantages as well as obstacles to its implementation. We recognize that adoption of new tools will likely require additional resources that may not be currently available to public health agencies or academic researchers and that obtaining necessary resources may require concerted and combined efforts of state/federal health agencies and academic research institutions. However, the addition of one or more of these tools to the current armamentarium may help in advancing our ability to detect clusters and improve our understanding of etiology of disease clusters. 

### 4.1. Rapid Case Ascertainment

As mentioned previously, an important barrier to cluster investigations is the time lag between diagnosis and complete enumeration of cases in cancer registries, potentially resulting in a missed possible cluster that would only be detected when all reporting for that time period is complete. This time lag could be avoided or minimized though rapid case ascertainment (RCA) methods whereby initial information about newly diagnosed cases is obtained through expedient transmission of pathology reports [59]. The modern RCA systems such as ePath collect electronic pathology reports and notify registry personnel or eligible researchers about new cancer cases with very little delay thereby allowing continuous assessment of cancer occurrence [60]. More recently developed approaches take advantage of the ePath technology by using natural language and knowledge-based processing to identify relevant tumor information in free text pathology reports. Software performing these tasks is currently being tested at several cancer registries [61]. Although modern RCA methods could play an important role in cluster investigations, their full integration into day-to-day cancer surveillance practices will likely take several years. Meanwhile, improving timeliness and completeness of cancer registration should be emphasized [60,62] by utilizing informatics technology including RCA methods and matching with relevant and evolving medical record databases.

### 4.2. Reconstructing Residential History

As nearly all cancers have protracted latency, current address, which is readily ascertained from the registry data, may be less important than residential history. Until recently, this presented a nearly insurmountable methodological limitation of cluster investigations, which had to use interviews to account for residential mobility. In recent years, however, residential history data have become increasingly available through population directories. Many of these directories are commercially available and could be used to construct residential histories during a cluster investigation. 

One recent study assessed the accuracy of residential histories in a population directory from LexisNexis, Inc. (Miamisburg, Ohio, USA). The analysis compared residential histories recorded in the LexisNexis directory to information collected from written surveys in a case-control study of bladder cancer in Michigan. The lifetime addresses obtained from LexisNexis and those reported in the surveys matched for 71.5% of participants [63]. The authors concluded that while higher accuracy is desirable, the availability of residential history from population directories such as LexisNexis represents a “vast improvement over the assumption of immobile individuals currently used in many spatial and spatiotemporal studies”.

### 4.3. Application of Spatial Statistics

Traditional approaches of working through the steps of cluster investigations [28] involve assessing rates for administrative geographical units such as ZIP codes or census tracts. An alternative approach is to examine clustering of disease in time and space untethered to pre-defined geographic units. This methodology was first suggested more than two decades ago [64], but computational and data management barriers at that time were formidable. Modern computer technology, however, enabled rapid developments in geospatial statistics and the practical applications of new methods of identifying and investigating clusters of cancer and other diseases. A number of currently available global clustering statistical tests are aimed at evaluating presence or absence of “hot spots of disease” on the map [64,65,66,67,68]. These tests, all based on the null hypothesis of “spatial randomness” have been reviewed in detail previously [69]; most were found to perform well, but depend heavily on the underlying assumptions. An example of a practical application of these global clustering tests is the recent analysis of brain cancer mortality in the USA, which demonstrated that brain cancers were more common in parts of Arkansas, Mississippi and Oklahoma, but found no specific localized clusters [70].

The use of tests of spatial randomness without an a priori expectation may be viewed as an advantage because performance of a test can be assessed in on its own merit; or a disadvantage because, as noted in a review by Kulldorff *et al.*, the findings of statistical analyses “may or may not correspond to true and interesting geographic patterns of the disease” [69]. Lawson proposed addressing this issue by applying a Bayesian approach that first incorporates a priori distribution for the study area and time of interest based on a pre-existing concern, and then performs a statistical assessment using one of the clustering tests [71,72]. 

As statistical methods for evaluating spatial patterns of health conditions and risk factors continue to develop [73]; their refinement presents a number of practical challenges. For example, it is important to keep mind that enhanced granularity of spatial data may require new ways of protecting confidentiality [74]. 

### 4.4. Continuous Monitoring of Registry Data

Although cluster analysis is not used in all registries, it could potentially be incorporated into routine practice assuming sufficient staff training and allocation of resources. In terms of feasibility, some state-based cancer registries use software such as SaTScan^™^ to verify community-reported cancer clusters and to find hot spots of late stage disease and other indications of screening and treatment need for selected cancers. Conducting constant monitoring could enable these agencies to quickly detect and investigate cancer clusters regardless of whether community members also report the same cluster and to perform descriptive epidemiologic studies that identify geographic aggregation of certain malignancies (see for example, [75]). Proactive scanning, even on a daily basis, is commonplace in influenza surveillance or in monitoring of asthma attacks, *i.e.*, conditions that are common, and develop relatively quickly following rapid changes in the environment. By contrast, monthly or even yearly proactive scanning presents a much greater challenge in cancer surveillance because the true changes in cancer occurrence are relatively slow and because small numbers of cases observed in a limited geographic area tend to produce incidence estimates that are unstable and difficult to interpret.

Continuous monitoring is not without limitations. One issue is the potential obligation for public health agencies to investigate and communicate findings of all software-identified cancer clusters. This obligation may overwhelm sparse public health resources at some agencies. A second issue is the need to verify whether data mining methods are up to the task of cancer cluster identification (e.g., can data mining be used to address the previously mentioned lack of historical residential data?). Lastly, spatial uncertainty must be addressed. Spatial uncertainty is the lack of, or the error in, knowledge about geographical position such as patients’ addresses that include P.O. boxes or rural routes (these are known to mischaracterize geographic location). It is also unclear how that uncertainty affects any association between environmental exposures and disease [76,77,78]. Further, research on how to visually display the extent of uncertainty is needed [79]. Although geographic information systems are becoming increasingly sophisticated in terms of addressing this issue, more research is needed to improve statistical methods and spatial data collection and quality control [80]. Thus, before state health departments embark on proactive monitoring for cancer, researchers need to verify that this approach has utility, given issues of latency and mobility, multiple comparisons, and temporal instability caused in part by small numbers. Issues associated with potential harm due to false positives as well as communication and ethical issues must also be evaluated. 

### 4.5. Improved Utilization of Electronic Data Sources

The linking of cancer cluster information with other forms of now rapidly digitalized health data [81] such as the electronic health record [82], population characteristics, and health care resources [83,84,85] in real-time could be helpful in pinpointing potential causal agents. 

A key concern is patient privacy; individuals must usually provide consent before such a linkage could occur. Alternatively, linkages could be performed with de-identified personal and geographic data. Current technical and practical barriers that would need to be resolved include incompatible data sets, lack of data standards, and data quality/integrity concerns. In order to resolve conflicts between data sets, researchers could utilize tools currently employed in software engineering to document digital processes (e.g., modifications in data formats and structure) and to track and ensure data integrity when consolidating multiple data sources. 

Because reports of residential cancer cluster investigations emanate from a residential network, another novel opportunity would involve harnessing the social network for data gathering on exposures and lifestyles. Development of a common interview, which could potentially be administered over the internet or mobile devices, would allow its rapid dissemination to members of the residential network. This interview could be customized to examine topics of particular relevance to each cluster, including the environmental issues of greatest concern. Linking the common interview questions across multiple potential cancer clusters may identify commonalities that would be missed when each is evaluated alone. This strategy would also help address the common problem of sparse data, which often plagues residential-based cancer cluster investigations. Members of the affected residential network could be enlisted to aid with customization of the common interview and with data collection (e.g., by linking residents to the interview and/or by directly collecting data via mobile devices). 

Information about clusters may be indirectly ascertained from digital social networks, which could shed light on individuals’ lifestyles and behavior from their interactions on these digital networks [86]. Kosinski *et al.* [87] demonstrated how preferences captured in Facebook (“likes”) predict behaviors of clusters of a social network, such as alcohol intake, smoking status, and narcotic use. For example, the more an individual “likes” to drink alcohol, the higher the probability that that individual’s social network also prefers that behavior. Hurdles that must be overcome in order to use this type of approach include obtaining consent from entire networks (as a coherent whole) and addressing the proprietary nature of these data. Information derived from these services needs to be validated for its utility in public health surveillance. 

### 4.6. Advances Using Biomarkers of Carcinogenesis

Traditional case-control studies of cancer clusters are problematic in part because of small sample sizes and the inability to control for confounders [88]. However, novel study designs that take into account new appreciations of the biological or “natural” history of cancer as a disease may help facilitate future investigations. Cancer is understood to evolve over a period of years or decades, with each new characteristic induced by multiple genetic and epigenetic changes. This concept originated with the recognition of the stepwise morphologic and genetic evolution of colon cancer [89]. Now many cancer sites have been described in exquisite genomic and epigenomic detail, with documented sequential progression of disruptions in the normal cell physiology [90]. This progression can be highly variable. Most age-related epithelial cancers have long latencies with more than five genetic mutations required. By contrast, childhood and therapy-related cancers (such as those that are related to the *MLL* gene) require only a small number of genetic changes and may have latencies of only several months [91,92]. Interestingly, any given population will harbor some persons carrying pre-cancerous cells; indeed, all individuals harbor some mutations that can contribute to cancer if more mutations occur [93,94]. Space-time clusters of cancer are likely to be related to causal factors that put an entire community at risk, but only impact cancer incidence in those at-risk individuals that have precancerous cells at the verge of becoming tumorigenic. Thus, a cause of the cluster may trigger the disease in only a small number of cases even though many individuals were exposed. This consideration underscores the need for alternative endpoints that are associated with increased risk of cancer (i.e., biomarkers of risk), but are detectable prior to tumor occurrence [95]. The relevant biomarkers available as endpoints can reflect genetic, epigenetic or RNA related changes as well tissue-based differences in protein levels. 

The use of biomarkers as endpoints has been advocated for cancer prevention trials [96], but could also apply to evaluation of clusters or effects of environmental exposures. Before these biomarkers are used in population studies, however, they need to be validated against clinically meaningful outcomes to avoid misinterpretation of results. 

### 4.7. Novel Concepts for Grouping Cases

With a trove of longitudinal clinical examinations and measures increasingly available in the health record, clinical characteristics of cases to be included in clusters could be better defined. For example, routine characterization of myeloid leukemias has evolved from the French-American-British Classification, which relied primarily on morphologic features, to the 2001 World Health Organization (WHO) classification which recommended cytogenetic assessment, to the 2008 WHO classification, which combines morphologic, cytogenetic, and molecular analyses [97]. Most recent clinical recommendations for the management of acute myeloid leukemia (AML) in children and adolescents indicate that an AML diagnostic workup should include at a minimum “morphology with cytochemistry, immunophenotyping, karyotyping, FISH (fluorescent *in situ* hybridization), and specific molecular genetics in the bone marrow” [98]. This example indicates that information on molecular characteristics of tumors is becoming increasingly available in the medical records and therefore can be used in cancer cluster investigations. The current SEER (Surveillance, Epidemiology and End Results) coding system (ICD-O-3) includes the most relevant cytogenetic and morphologic criteria and simply adopting this coding scheme will help to incorporate the most pertinent and specific diagnostic details in a systematic fashion [99].

It is becoming increasingly clear that many cancer types can be subdivided into entities based on molecular characteristics that may have distinct etiologies, prognoses, and responses to therapies [100]. Molecular markers of tumors are increasingly being incorporated in routine practice to establish cancer progress or guide treatment; cancer registries are beginning to find ways to capture these data, as well [100]. Molecular information could be collected from medical records of individuals within putative cancer clusters and be used to classify cases into more homogenous subgroups for analysis; this has the potential to be useful in uncovering etiologic factors that are relevant to only certain of the cancer subtypes. For example, triple negative breast cancer has some shared and some different risk factors compared to other forms of breast cancer [101]. 

In addition, biomarkers can be used in cancer cluster investigations to identify tumors with similar molecular characteristics that may share a common cause. Our current method of classifying cancer by primary site (e.g., organ) and/or broad histological type may insufficient for understanding cancer etiology. Cancer cells may share common characteristics regardless of cancer site [102] and common cellular pathways for growth and survival exist across multiple tissues. These characteristics include rapid cell growth, resistance to apoptotic signals, uncoupling of differentiation and cell division, and maintenance of the ends of chromosomes (telomeres). An example is the mutation of *TP53* or *RAS* genes which are mutated across cancers of the lung, colon, pancreas, blood, skin and other sites [103]. These common mutations in disparate cancers may have similar causes, for instance nucleophilic chemicals or aflatoxin [104]. Another example would be *IDH* mutations, common in leukemia, brain cancer, and cartilaginous tumors, and related to broad epigenetic patterning [105]. 

Therefore, it is possible that for cancer cluster investigations, cancers should be reclassified according to subtype within a major cancer type as well as according to their carcinogenesis features such as presence of mutations or epigenetic changes as opposed to location or appearance. For some cancers (e.g., pediatric leukemias), this type of data may already be available in medical records. For other types of cancers, data are currently being collected only for research purposes. 

### 4.8. Infection and Cancer Clusters: An Example of Pediatric Leukemia

Pediatric leukemia is a disease known to involve genetic aberrations that occur during distinct time periods: the first aberrations occur during pregnancy (prenatally) and subsequent aberrations occur postnatally [106]. Leukemia incidence is, at least to some extent, calendar time-dependent, although not unequivocally seasonal [107] and thus leukemia clusters are likely an expression of postnatal causal events which have impacted communities at about the same time. It is hypothesized that such causal events are likely to be infectious [108,109,110]. For example, flu epidemics are often followed by transient increases in leukemia rates [111]. Further, a widely publicized leukemia cluster in Niles, IL was reported to be “accompanied by the parallel appearance of rheumatic-like illness” in the same community, suggesting a common infectious etiology [112]. 

A more recent example of a potential infectious cause of leukemia is found in the description of the Fallon, NV cluster, which affected children from 2 to 19 years of age and included a range of common childhood leukemia diagnoses [113]. All leukemia cases occurred in the space of three years and most were restricted to one year [43]. With such a disparate age range and leukemia subtype diagnoses, the cluster is unlikely to be linked to cancer “initiating” events that occur prenatally [106]. The initiating mutations occurring earlier in the children’s life may have dissimilar causes and identities, leading to different subtypes of leukemia at different ages, despite disease diagnoses being tightly clustered in time. The epidemic appearance of the cluster only makes sense as a clustering of “secondary genetic events” precipitated by a new environmental stimulant such as infection, one that might have been introduced to the community from the town’s transient military population [43]. 

Similarly, an apparent cluster of seven cases of childhood acute lymphoblastic leukemia (ALL), which occurred over a four-week period in Milan, Lombardy, Italy, was associated with an outbreak of the AH1N1 influenza virus which occurred several weeks prior to the diagnoses [114]. The authors note that this is “compatible with the “delayed infection” hypothesis for childhood ALL in which an abnormal immune or inflammatory response to a common infection promotes ALL in susceptible individuals”. 

Infection is not the only potential cause for time-dependent clustering, as shown by other examples of leukemia clusters that may have been incited by chemical stimuli [115,116]. However, infection remains a viable theory in leukemia clustering (e.g., “population mixing” theories [117]), and the role of infection in leukemia and other cancers is currently under exploration using sequencing and discovery methods similar to that described for new emerging viral illnesses [118].

Considering that cancer clusters (if related to a common a cause) are likely to be a response to a proximate (in time) change in the environment and also are likely to be a rare response to a common factor, cluster investigations should focus on the identification of factors that have impacted the community at large rather than just the individuals who contracted cancer. The likelihood of success for this type investigation would be increased if it were performed immediately upon identification of a cluster. Such an investigation can compare a community with other communities that have not experienced similar health outcomes, and focus on agents that factor into the known etiology of specific cancer types. For example, Steinmaus *et al.* [119] examined the Fallon, NV cluster in this fashion by comparing the Fallon community with other communities of similar size in different locations that held military bases. 

For leukemia, infectious stimuli can be explored by reviewing hospital records and registry data to search for unusual co-occurrences of related health events prior to or concurrent to the cluster. Biological samples can be retrieved from cancer cases and community members (tumor and constitutive material) to test for specific hypotheses (infectious agents), or in the absence of specific tests more exploratory profiling of chemical and infectious exposures. Academic or industry laboratories that could help support such efforts should be recruited at early stages if possible. 

### 4.9. “Omics” Approaches

To deal with the complexity of multiple exposure factors that are difficult to study using existing methods, researchers have developed the concept of the exposome, which describes the “totality of environmental exposures” an individual encounters from birth to death [120]. The exposome concept was introduced as an analog to the genome, which encapsulates almost all of the hereditary information of an individual and consists of 3 billion chemical bases that encode about 20,000 genes. Genomic technologies are already used to examine clustering of disease. For example, Palacios *et al.* [121] discovered a novel pathogen in a cluster of patients who developed encephalopathies shortly after a solid organ transplant from a single donor. By applying high-throughput sequencing technology of samples from deceased patients, the investigators were able to isolate genetic material of the causal virus amongst a complex mixture of host microflora without any *a priori* knowledge of the infectious agent. Like the genome, studies of the exposome may be designed to query various combinations of environmental factors. Such studies may be possible after ascertainment of a “baseline” or “reference” exposome from population-based biomarker surveillance data [122,123]. Unlike the genome, however, the technology needed to ascertain an individual’s exposome is still in the conceptual stage. 

## 5. Conclusions

In this communication, we reviewed the challenges associated with successfully identifying community cancer clusters and their causes and described scientific advances—in various stages of maturity—that could potentially be harnessed to improve our ability to conduct community cancer cluster investigations in a way that might lead to a better understanding of cancer etiology. Following are key conclusions and recommendations:

1. The challenges to understanding why cancers may cluster in time and space were first enumerated several decades ago, but still limit investigations today.
2. While understanding the role of known or perhaps novel risk factors is an objective of cluster investigations, health agencies have a responsibility to the public to respond to community concerns. Interactions during a cluster investigation provide opportunities to bring to light a public health, environmental, social or other health problem as well as to educate an engaged group of citizens on the frequency, etiology, and prevention of cancer, as well as on exposure issues of concern.
3. Advances in our understanding of cancer development and cause, coupled with new methods of spatial statistics and novel technologies,, present opportunities for examining cancer clusters in novel ways and may lead to greater success in identifying cancer clusters and understanding cancer cluster etiology.
4. Technological advances may also improve the collection of information on residential history and population characteristics.
5. Biological advances can improve the use of biomarkers for understanding cancer etiology, for identifying and defining cases, and considering under-explored possible causes of cancer clusters such as infection.

The advances described here, including those that are in the early stages of development, will require a commitment of resources in order to bring these various approaches to fruition. While cluster investigations serve several purposes, public health protection related to cancer cluster investigations will ultimately derive from fundamentally improved methods for investigating those clusters. 
